# Mapping habitat suitability for Asiatic black bear and red panda in Makalu Barun National Park of Nepal from Maxent and GARP models

**DOI:** 10.1038/s41598-021-93540-x

**Published:** 2021-07-08

**Authors:** Huiyi Su, Manjit Bista, Mingshi Li

**Affiliations:** 1 College of Forestry, Nanjing Forestry University, Nanjing, 210037 China; 2 Co-Innovation Center for Sustainable Forestry in Southern China, Nanjing Forestry University, Nanjing, China; 3 Department of National Parks and Wildlife Conservation, Ministry of Forests and Environment, Babarmahal, Kathmandu, Nepal

**Keywords:** Biodiversity, Ecological modelling

## Abstract

Habitat evaluation is essential for managing wildlife populations and formulating conservation policies. With the rise of innovative powerful statistical techniques in partnership with Remote Sensing, GIS and GPS techniques, spatially explicit species distribution modeling (SDM) has rapidly grown in conservation biology. These models can help us to study habitat suitability at the scale of the species range, and are particularly useful for examining the overlapping habitat between sympatric species. Species presence points collected through field GPS observations, in conjunction with 13 different topographic, vegetation related, anthropogenic, and bioclimatic variables, as well as a land cover map with seven classification categories created by support vector machine (SVM) were used to implement Maxent and GARP ecological niche models. With the resulting ecological niche models, the suitable habitat for asiatic black bear (*Ursus thibetanus*) and red panda (*Ailurus fulgens*) in Nepal Makalu Barun National Park (MBNP) was predicted. All of the predictor variables were extracted from freely available remote sensing and publicly shared government data resources. The modeled results were validated by using an independent dataset. Analysis of the regularized training gain showed that the three most important environmental variables for habitat suitability were distance to settlement, elevation, and mean annual temperature. The habitat suitability modeling accuracy, characterized by the mean area under curve, was moderate for both species when GARP was used (0.791 for black bear and 0.786 for red panda), but was moderate for black bear (0.857), and high for red panda (0.920) when Maxent was used. The suitable habitat estimated by Maxent for black bear and red panda was 716 km^2^ and 343 km^2^ respectively, while the suitable area determined by GARP was 1074 km^2^ and 714 km^2^ respectively. Maxent predicted that the overlapping area was 83% of the red panda habitat and 40% of the black bear habitat, while GARP estimated 88% of the red panda habitat and 58% of the black bear habitat overlapped. The results of land cover exhibited that barren land covered the highest percentage of area in MBNP (36.0%) followed by forest (32.6%). Of the suitable habitat, both models indicated forest as the most preferred land cover for both species (63.7% for black bear and 61.6% for red panda from Maxent; 59.9% black bear and 58.8% for red panda from GARP). Maxent outperformed GARP in terms of habitat suitability modeling. The black bear showed higher habitat selectivity than red panda. We suggest that proper management should be given to the overlapping habitats in the buffer zone. For remote and inaccessible regions, the proposed methods are promising tools for wildlife management and conservation, deserving further popularization.

## Introduction

Habitat is the type of natural environment in which a particular species lives or can find food, shelter, protection, and mates for reproduction. Knowledge of habitat preference and geographical distribution is essential for the conservation of threatened species^1,2^.

In recent years, several statistical and computer-based methods have been utilized to map biological and ecological data and to spatially interpolate species distributions and other bio-spatial variables of interest. Many fields of research rely on predictive models for assessing patterns of species distribution^3,4^. Recently, with the development of GIS, Remote Sensing, and GPS techniques, multiple models have been used to assess suitable habitat distribution, including mechanism models^5^, regression models^6^, and ecological niche models^7,8,9,10^. Mechanism models do not need species distribution point data, but establish the corresponding evaluation criteria according to the influence of habitat factors on species distribution, so as to simulate the suitable habitat of a species. However, mechanism models have some limitations because they do not consider the accessibility of the habitat, and are subjective in the classification and weight determination of factors. With field observations, the absence of animal traces in a certain place does not mean that the animals have never appeared there. Compared with regression models, ecological niche models only need the animals "presence points" and do not need "non-presence points” data. Among the ecological niche models, Maxent and GARP have often shown accurate prediction capabilities in simulations and evaluations with presence-only data, outperforming classical modeling approaches, such as domain, bioclim, and logistic regression^11,12,13^. Most Species Distribution Modeling (SDMs) are based on correlation statistics and cannot strictly infer causality, but the summation of correlation results based on ecologically significant predictors can support hypotheses^14^. Many SDMs include a large number of candidate predictors, motivated by the availability of the data set, friendly presentation in a statistical package, and the ability of the model to identify important variables in those predictors^15^. Some modelers argued that the success in interpreting habitat suitability distribution also depends on the correct selection of environmental variables used in the SDM model^16^.

Maxent is a software for modeling species niches and distribution that applies a machine learning technique called maximum entropy modeling, which is for modeling geographic distributions of a species based on the ecological niche theory proposed by Jaynes^17^. This method was initially employed to estimate the density of presence across the landscape^9^, relying on information from species presence data to explore the possible distribution of a target species within a study area. Now it is widely used in SDM^18,19^. The Genetic Algorithm for Rule set Production (GARP) is a common and flexible species distribution modeling tool, which is based on a genetic algorithm that develops sets of rules to constrain the species distribution^20^. It generates a random set of mathematical rules following an iterative process of rule selection, through testing, merging, and denying^21^. These sets of rules are combined in a random way to generate the potential niche of the species under the environmental conditions. GARP has been applied to studies that attempt to predict the risk of biological invasions based on the potential geographic distribution of species in native and non-native habitats^22,23^. Even so, the prediction accuracy and performance of individual SDMs vary widely among methods and species^24^. The integrated approach of multi-individual models provides robust estimates of potential species’ distributions^25,26^. Ensemble maps that highlight areas of consistency among different model predictions offer a way to reduce the uncertainty of results based solely on one SDM model^8^.

The Asiatic black bear (*Ursus thibetanus*) and Himalayan red panda (*Ailurus fulgens*) are two threatened species that are recorded in Makalu Barun National Park (MBNP) along with other areas of Nepal. Previous studies related to the distribution, diet, habitat, and threats of the Asiatic black bear and red panda have examined the two species separately. These species, however, live in habitats that have similar altitudinal ranges^27–30^ and are sympatric in many protected areas of Nepal including MBNP^31^. Both species are listed in Appendix I of the Convention on International Trade in Endangered Species of Wild Fauna and Flora^32,33^. The Asiatic black bear, which is native to 20 Asian countries, including Nepal, is registered as vulnerable on the International Union for Conservation of Nature red list^34^. The Asiatic black bear of Nepal favors mixed temperate oak (*Quercus semecarpifolia*) forests^35^. In central Nepal, it has been recorded between the altitudinal range from 1600 to 3200 m^29^, however in some regions, the preferred elevation of Asiatic black bear is between 2500 and 3000 m, and its altitudinal limit is 4300 m^34,36^. The Himalayan red panda is distributed in Nepal, Bhutan, India, Myanmar, Tibet and the western Yunnan Province of China^37,38^, and also Laos^33^. It is a protected mammal by the National Parks and Wildlife Conservation Act of Nepal (1973), and is listed as endangered on the International Union for Conservation of Nature (IUCN) red list. The red panda prefers temperate evergreen forests where bamboo is the dominant ground cover, due to the leaves and young shoots of bamboo being its primary food source^39–42^. Although red panda is a protected species by national and international laws, its population is in decline due to anthropogenic pressures and habitat fragmentation^43^.

Studies related to the diversity of these two species in the same habitat are lacking, resulting in a gap between research and local conservation actions, especially in Nepal. Due to the similarity in their habitat, distribution, diet, and threats, it is beneficial to identify the overlapping spatial habitat between these species and to identify indicative habitats to focus conservation efforts^25,26^. Such efforts would allow the park authority and conservation partners to engage in those areas of MBNP that are favorable for the target species, allowing for the simultaneous conservation of the two threatened fauna. Such evaluation constitutes a footstone in wildlife protection and management, offering a scientific rationale for the improvement of conservation policies^44^.

Understanding the distribution of Asiatic black bear and Himalayan red panda habitat in MBNP is important for the improvement of research outcomes and conservation plans. Thus, the main objective of this study was to: (1) predict the suitable habitat for Asiatic black bear and red panda in Makalu Barun National Park based on presence data and a range of environmental variables by using Maxent and GARP; (2) compare the results of two models for the individual species and determine the extent of the overlapping suitable habitat of these two species in the study area; and (3) propose recommendations and analysis for the conservation of the two species in MBNP.

## Material and method

### Study area

**Figure 1:**
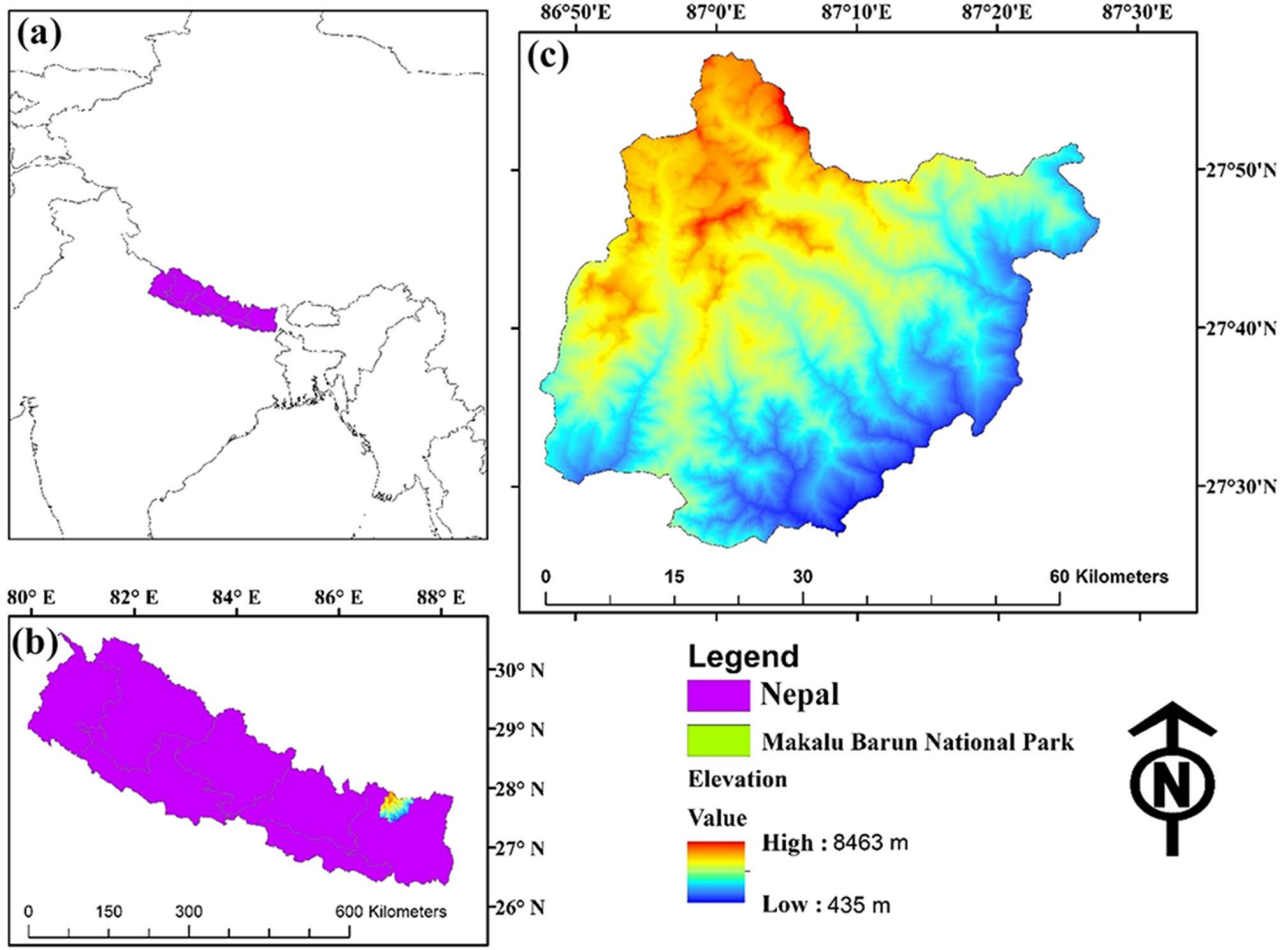
Location of the study site. (**a**) Location of Nepal in Asia, situated between China in the north and India in the south. (**b**) Location of MBNP in Province 1 in Nepal. (**c**) The elevation map of MBNP. Map created in ArcMap 10 of the Environmental System Resource Institute, Inc. (https://desktop.arcgis.com/zh-cn/arcmap/).

Makalu Barun National Park (MBNP) was established in 1991, with an area of 1500 km^2^ (Fig. 1). To meet the needs of local people and to minimize human-wildlife conflict, an area of 830 km^2^ was established as a buffer zone (BZ) around the park in 1999^45^. MBNP extends from an elevation of lower than 435 m in the South up to 8,463 m (peak of Mt Makalu) in the North within an areal distance of 40 km. This vertical relief is more narrow than any other conservation unit on earth and is located at the near-tropical latitude. This park possesses tremendous bioclimatic amplitude and a corresponding broad range of ecological community types^46^. The bioclimatic zones encompassed in this park range from the upper tropical to the nival, and there are 20 types of ecosystems with 12 types of vegetation^47^.

There are seven major river systems in the park, among which 5 are the tributaries of the Arun River. The monsoon rainfall in the study area ranges from 1000 to 4000 mm per year^48^. This park also attracts around 1000 tourists annually and their destinations are usually Mt Mera and Mt Makalu. Approximately 35,000 people reside in the villages within the buffer zone and most of them belong to ethnic communities (Rai, Sherpa, Bhote). Their primary economic activities are agriculture, animal husbandry, and seasonal works (like tourism, trade).

### Species presence data

**Figure 2:**
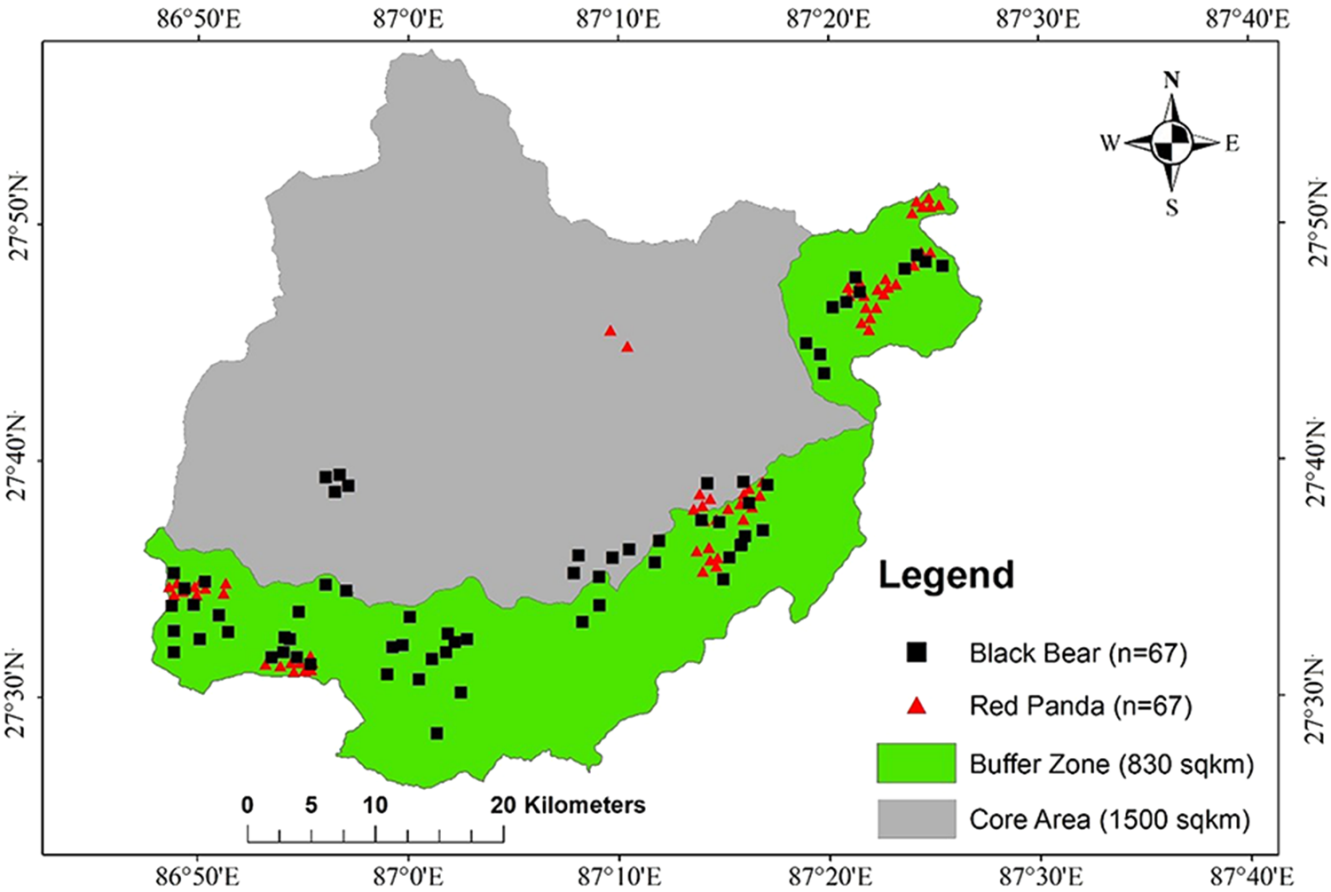
Presence points of the species in the study area. Map created in ArcMap 10 of the Environmental System Resource Institute, Inc. (https://desktop.arcgis.com/zh-cn/arcmap/).

We used data obtained by Bista et al. during field visits with MBNP staff from May 2015 to June 2016^49^. They conducted informal interviews with local people and staff of the national park to identify potential habitats of the Asiatic black bear and red panda within the park and its buffer zone. Presence points were recorded with a GPS based on observations of the species and the finding of the species’ scat. The sample size has an essential influence on the output of the species distribution models because the accuracy and stability of the two models increase with the sample size^50^. For this study, we used 67 presence points for each species and their locations are represented in Fig. 2.

### Data and processing

The input to the model was a series of variables and the land cover map obtained by analyzing remote sensing data. Variables included topographic, bioclimatic, vegetation-related, and anthropogenic variables.

1. To prepare the land cover map, an image collected by Landsat 8 OLI was downloaded from the United States Geological Survey (USGS, https://glovis.usgs.gov/). The resolution of the image is 30 m with a path/row number of 140/041. The image was already ortho-rectified and also atmospherically corrected by USGS at our request when placing the data order. Because of seasonal constraints like atmospheric haze, monsoonal cloud cover, and snowfall, late autumn is the most optimum season for remote sensing data acquisition for land cover mapping in Makalu Barun National Park^51^. The image acquisition date used in this study was November 11, 2016. An image subset for the study area was clipped from the Landsat 8 scene by using a vector shapefile (administrative boundary) obtained from the Department of National Park and Wildlife Conservation. The support vector machine (SVM) classifier has been widely adopted for land-cover classification^52^, and we selected it for land cover classification in the current work.
2. For the topographic variables, elevation, aspect, and slope were used as they are the most critical topographical factors impacting habitat selection by terrestrial animals. A 30-m resolution digital elevation model (DEM) from the Japan Aerospace Exploration Agency’s (JAXA) (https://www.eorc.jaxa.jp/ALOS/en/aw3d30/data/index.htm) was used to calculate slope and aspect. And the resampling tool was subsequently employed to convert the spatial resolution of the elevation, aspect, and slope to 100 m.
3. Necessary bio‐climatic variables (annual time series with annual means, seasonality, and extreme or limiting temperature and precipitation) were downloaded from the WorldClim historical database (http://worldclim.org/). Version 2.0 of this database provides a set of 19 global bio-climatic variables derived from over 4,000 weather stations, which are averaged between the years 1970 and 2000, and with a spatial resolution of approximately 1 km (i.e. 30 s)^53^. The bio-climatic information represented by this dataset was considered to be meaningful at the time of acquisition of the presence points, assuming that no drastic disasters had occurred in the study area. For this study, the spatial resolution of all 19 bio-climatic variables was resampled to 100 m using ArcGIS. Specifically, we used the Inverse Distance Weighted (IDW) algorithm in geostatistics to interpolate the station’s observations to create bio-climatic raster images first, followed by resampling them to 100 m resolution.

Removing highly correlated variables for species distribution modeling is recommended for reliable and unbiased output^54^. We statistically tested the correlations among all the 19 bioclimatic variables using the presence and background locations through examining the Pearson correlation coefficient values (the absolute value greater than 0.75). Both Ecological Niche modeling Tools (ENM Tools 1.4.4) and Principal Component Analysis were used within ArcGIS to remove highly correlated predictors. The two factor-screening methods produced similar results, and out of 19 variables, only six of them were kept for use (Table 1).

**Table 1:** Variables selected for modeling.

Category	Variables	Abbreviation	Unit	Source
Bioclimatic variables	Annual Mean Temperature	bio1	Degree C	WORLDCLIM
	Mean Diurnal Range (Mean of monthly (max temp − min temp))	bio2	Degree C	
	Isothermality (BIO2/BIO7) × 100	bio3	Percentage	
	Precipitation of Driest Month	boi14	mm	
	Precipitation of Driest Quarter	bio17	mm	
	Precipitation of Coldest Quarter	bio19	mm	
Topographic	Elevation	Elev	m	JAXA
	Aspect	Aspect	Degree	
	Slope	Slope	Degree	
	Distance to water	dis-water	m	GEOFABRIK
Vegetation Related	Forest Cover	FC	Percentage	Global Forest Change
Anthropogenic	Distance to Settlement	dis-sett	m	DNPWC
	Distance to path	dis-path	m	GEOFABRIK
Land cover	Catalogs of land cover	LC	–	By calculation

(4) Forest is a major component of an animal’s habitat. In this study, the tree canopy cover from Global Forest Change(https://earthenginepartners.appspot.com/science-2013-global-forest/download_v1.5.html) was used to model the habitat of the target species. Tree canopy cover was defined as the canopy closure for all vegetation (broadleaved or conifers) taller than 5 m in height, which is encoded as a percentage per output grid cell in the range 0–100. The full range map was reclassified to 5 grades or levels with an equal interval of 20, which was later resampled to 100 m resolution.

(5) Along with topographic and vegetation-related variables, anthropogenic variables are equally important to identify how human activities affect the distribution of wild animals. The shapefile of paths and settlements inside the study area was downloaded from the Geofabrik (http://download.geofabrik.de/asia/nepal.html) and then rasterized. Of which, locations of settlements were made available by the Department of National Parks and Wildlife Conservation (DNPWC) and Department of Survey, Nepal.

### Species distribution Modeling

This study employed Maxent and GARP, to predict the suitable habitat of *Ursus thibetanus* (Asiatic black bear) and *Ailurus fulgens* (Himalayan red panda) in Makalu Barun National Park.

We used Maxent version 3.4.1^55^ (http://biodiversityinformatics.amnh.org/open_source/Maxent/) to model suitable habitat of both Asiatic black bears and red pandas in the study area. Though Maxent is competent at making robust predictions with default parameters without much effort in parameter tuning^56,57,58^, the users must make several decisions from a wide variety of settings in the software package to build models from their data^54^. The species presence points and 13 environmental variables (Table 1) described above were used as model inputs. At least a 500 m distance between species presence points was maintained to reduce spatial autocorrelation. Ten replicates were specified, and 1000 maximum iterations were conducted during the modeling^59^. A Jackknife test was used to examine the importance of individual variables for Maxent predictions. The regularized training gain describes the improvement of the model distribution that fits presence data compared to a uniform distribution. The Jackknife test gives training, testing, and regularized training gains for three scenarios (without variables, with only one variable, and with all variables) for different environment variables used for prediction. Furthermore, a threshold rule of 10 percentile training presence was applied.

The same 13 variables used for Maxent were used to predict the suitable habitat using Desktop GARP version 1.1.6. The Dataset Manager from GARP was utilized to build the dataset for modeling with the mentioned variables above. All the presence points of both species were split into ten randomly selected iterations, with 75% of the dataset used for training and 25% used for testing^8^. The training datasets were used in model building and the testing datasets were used to compute model accuracy metrics^60,61^. We used 200 model runs with 1000 maximum iterations and a coverage limit of 0.01. The best subset selection was active with an extrinsic omission measure and a 10% hard omission threshold. 20 models were under the hard omission threshold and the commission threshold was 50% of the distribution^62^. The top ten subsets models for both species were summated with Desktop GARP to assess model agreement and accuracy. Model accuracy metrics for each GARP experiment were calculated from the testing dataset (25%) withheld from the model building process. Among all outputs, 50% of the models were chosen at the subset of each species, among them the one with the highest test accuracy and lowest omission (i.e., best model) was selected as the most probable model to estimate suitable habitat distribution of both species^20^.

After running the models using all the variables described above, the continuous habitat suitability map was converted to a suitable/unsuitable binary map for both species. The suitable habitat maps of both species were then overlaid over the land cover map to determine the extent of suitable habitat for each species individually. Subsequently, the maps showing the suitable habitats of both species by the same model were overlaid to delineate the overlapping habitat of the two species.

### Accuracy assessment

Accuracy assessment is essential to validate the models and to understand their performances.

For the land cover classification, we implemented a simple random sampling strategy to evaluate the accuracy of our classifications. 150 random points in the study area with a minimum distance of 1000 m were created with ArcGIS, and the classification attribute of each point location was compared with the visual interpretation results of the corresponding high resolution Google Earth reference maps. Thus, a confusion matrix was constructed to derive the overall accuracy, producer’s accuracy, user’s accuracy, and Kappa coefficient, to represent the reliability of classifications.

**Figure 3:**
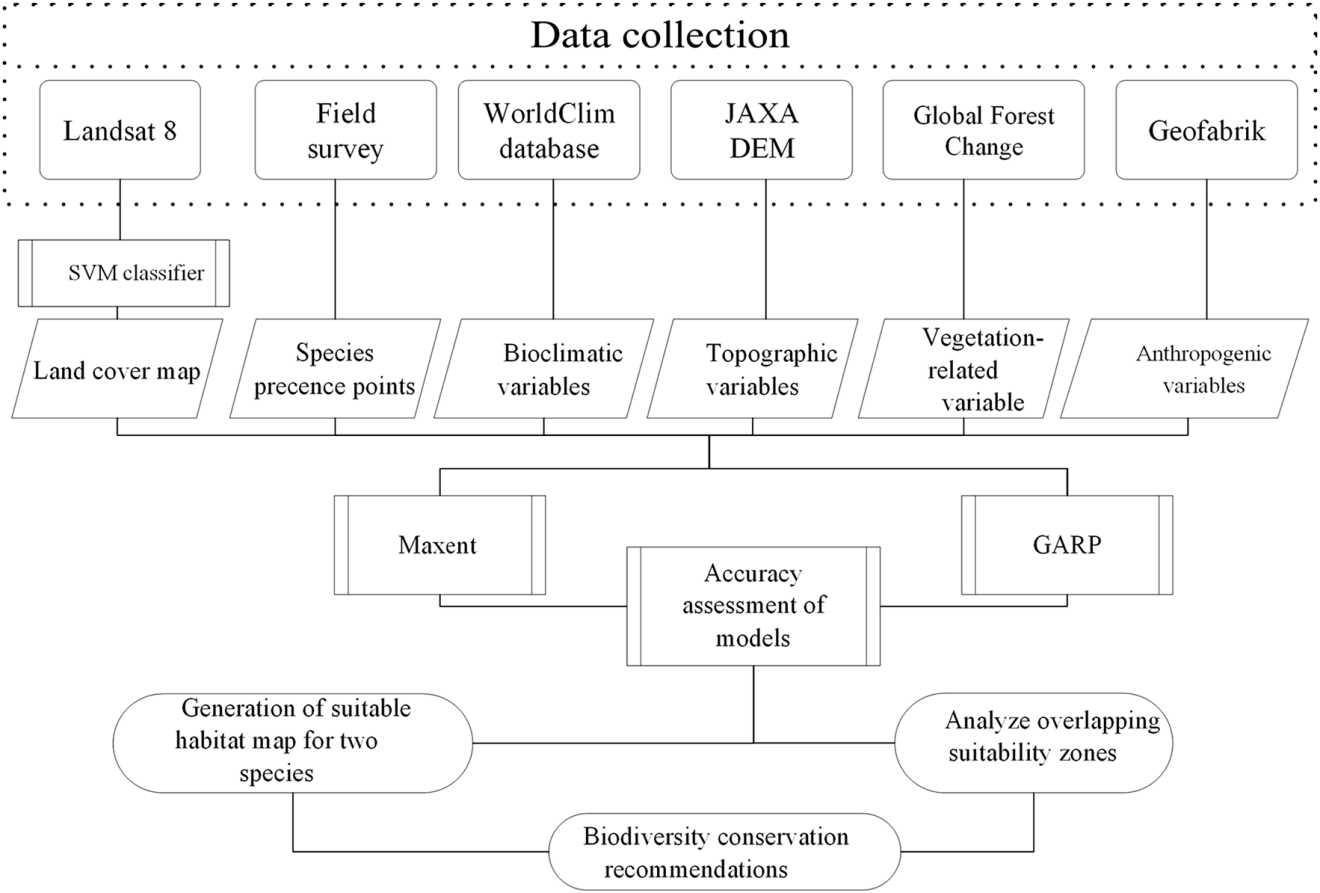
Research framework of the study.

The SDM models were evaluated by measuring the area under the receiver-operator curve (AUC) of the models. AUC is a widely used procedure for comparing species distribution model performance^9,63,64,65^, which is a threshold independent measure of predictive accuracy based only on the ranking of locations. AUC is interpreted as the probability that a randomly chosen presence location is ranked higher than a randomly chosen background point^54^. And the AUC value ranges from 0 to 1, the closer the value of the AUC to 1, the better the fit is of the model; where an AUC < 0.7 denotes poor model performance, 0.7–0.9 denotes moderately useful model performance, and > 0.9 denotes excellent model performance^66^ (Fig. 3).

## Results

### Land cover mapping

**Figure 4:**
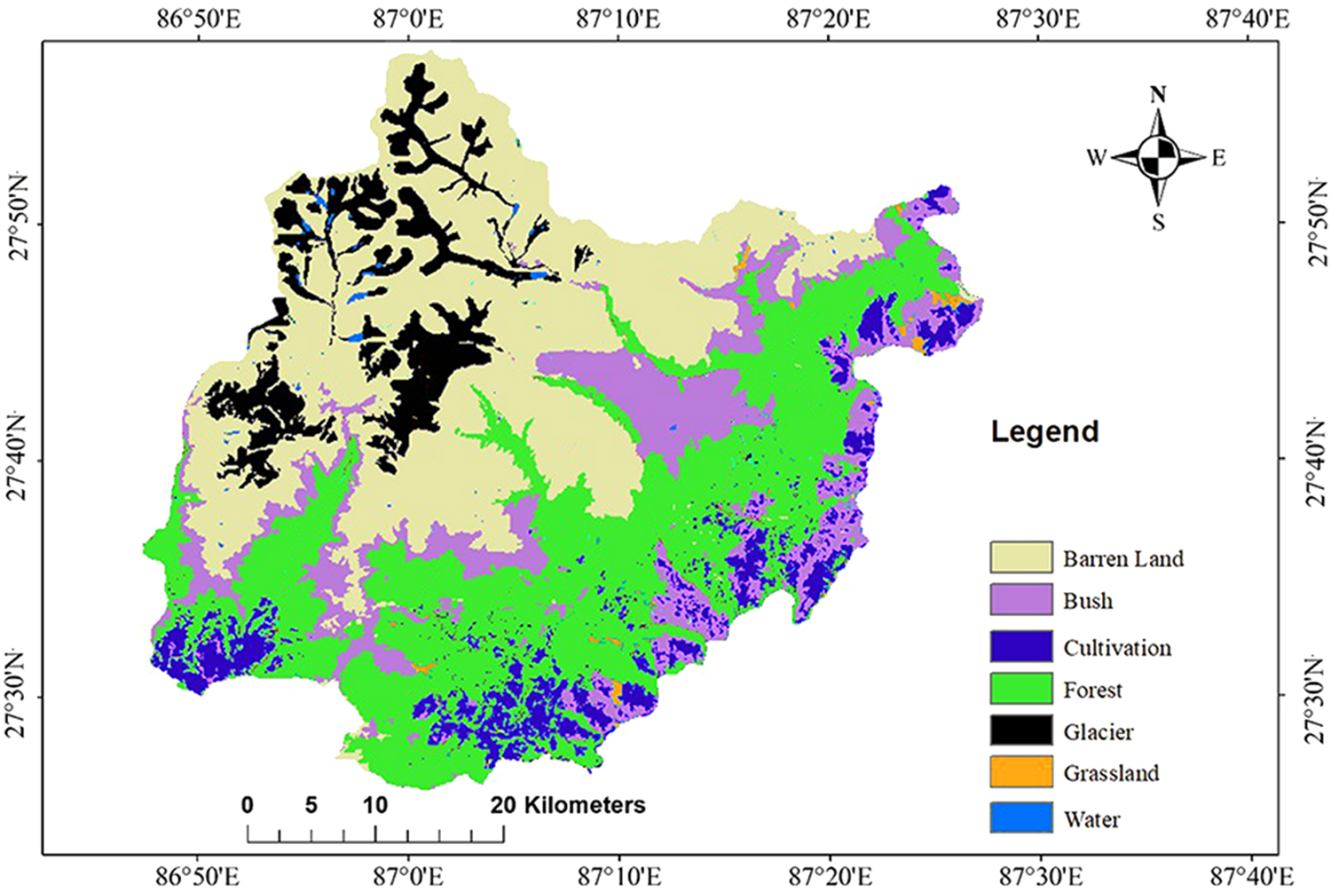
Land cover map of MBNP in 2016. Map created in ArcMap 10 of the Environmental System Resource Institute, Inc. (https://desktop.arcgis.com/zh-cn/arcmap/).

Figure 4 shows the land cover classification map. The overall accuracy, producer’s accuracy, user’s accuracy and Kappa coefficient were calculated to test the accuracy of land cover mapping. The independent validation by using the 150 points indicated that the user’s accuracy ranged from 83.3% to 100%, while the producer’s accuracy ranged from 50 to 100% for all the 7 land cover classes, and the overall accuracy was estimated at 88% with a Kappa coefficient of 0.84, showing a relatively substantial reliability of classification. The results of land cover exhibited that barren land covered the highest percentage of area in MBNP (36.0%) followed by forest (32.6%) (Table 2). The bushy area covered 14.8% of the total land in the study area, while cultivation covered only 6.8%. In the mountainous region of MBNP, 8.6% was enclosed by glaciers and water body coverage was only 0.5%.

**Table 2:** Area and percentage of the preferred habitat of black bear and red panda over different land cover categories.

Categories	Total area (km^2^)	% in MBNP (%)	Buffer zone area (km^2^)	Maxent				GARP			
				Black bear		Red Panda		Black bear		Red Panda	
				Suitable area (km^2^)	%	Suitable area (km^2^)	%	Suitable area (km^2^)	%	Suitable area (km^2^)	%
Barren Land	840.6	36.0	37.6	30.9	4.3	8.4	2.5	55.8	5.2	28.0	4.0
Bush	345.5	14.8	161.6	121.0	16.9	80.0	23.3	221.9	20.7	144.0	20.2
Cultivation	158.2	6.8	156.0	94.9	13.3	35.8	10.4	132.7	12.4	106.2	14.9
Forest	760.7	32.6	459.5	456.1	63.7	211.4	61.6	643.4	59.9	420.1	58.8
Glacier	200.7	8.6	1.4	1.8	0.2	0.5	0.1	2.6	0.2	2.4	0.3
Grassland	13.5	0.6	11.3	8.2	1.1	5.5	1.6	11.5	1.1	9.8	1.4
Water body	10.7	0.5	2.7	3.1	0.4	1.4	0.4	5.9	0.6	3.8	0.5
Grand Total	2330	100	830	716	100	343	100	1074	100	714	100

### Predicted suitable habitat

**Figure 5:**
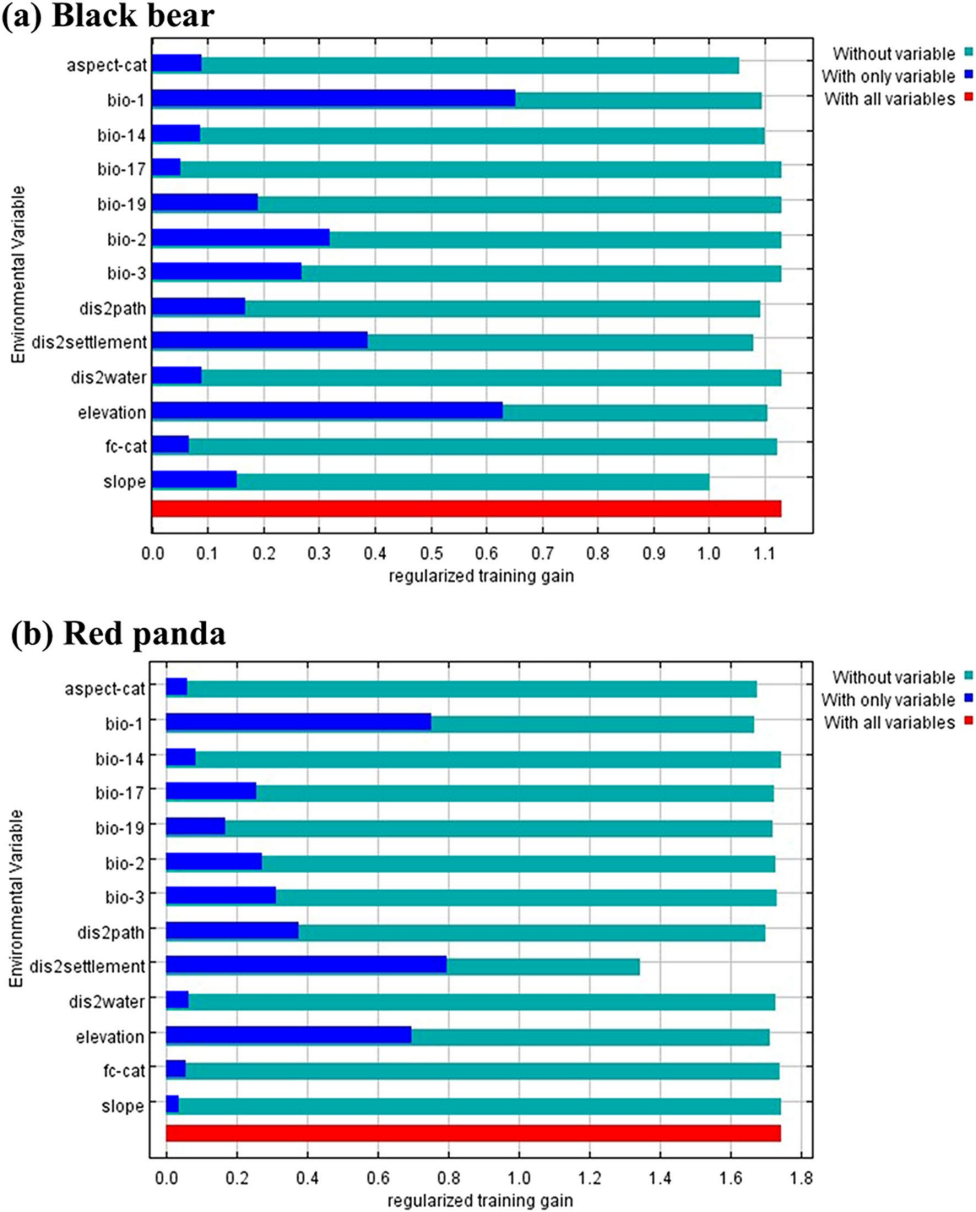
Jackknife test for regularized training gain of individual environmental variable importance (blue bars) relative to all environmental variables (red bar) for the Maxent model.

Figure 5 shows the relative importance of different environmental variables based on the results of the jackknife tests in Maxent. Analysis of regularized training gain showed only three out of the thirteen variables were considered important and contributed to the prediction of suitable habitat for both species in the study area. These variables were distance to settlement, elevation, and mean annual temperature. Among these three, the annual mean temperature provided the highest gain when used in isolation, which therefore, appears to have the most useful information by itself. The variables bio2, bio3, bio19, distance to path and slope had a moderate contribution to the prediction of suitable black bear habitat and the other remaining variables had a negligible contribution. For building the model of the suitable red panda habitat, bio2, bio3, bio19, distance to path and slope had a moderate contribution to the model, while the role of the remaining variables was negligible.

**Figure 6:**
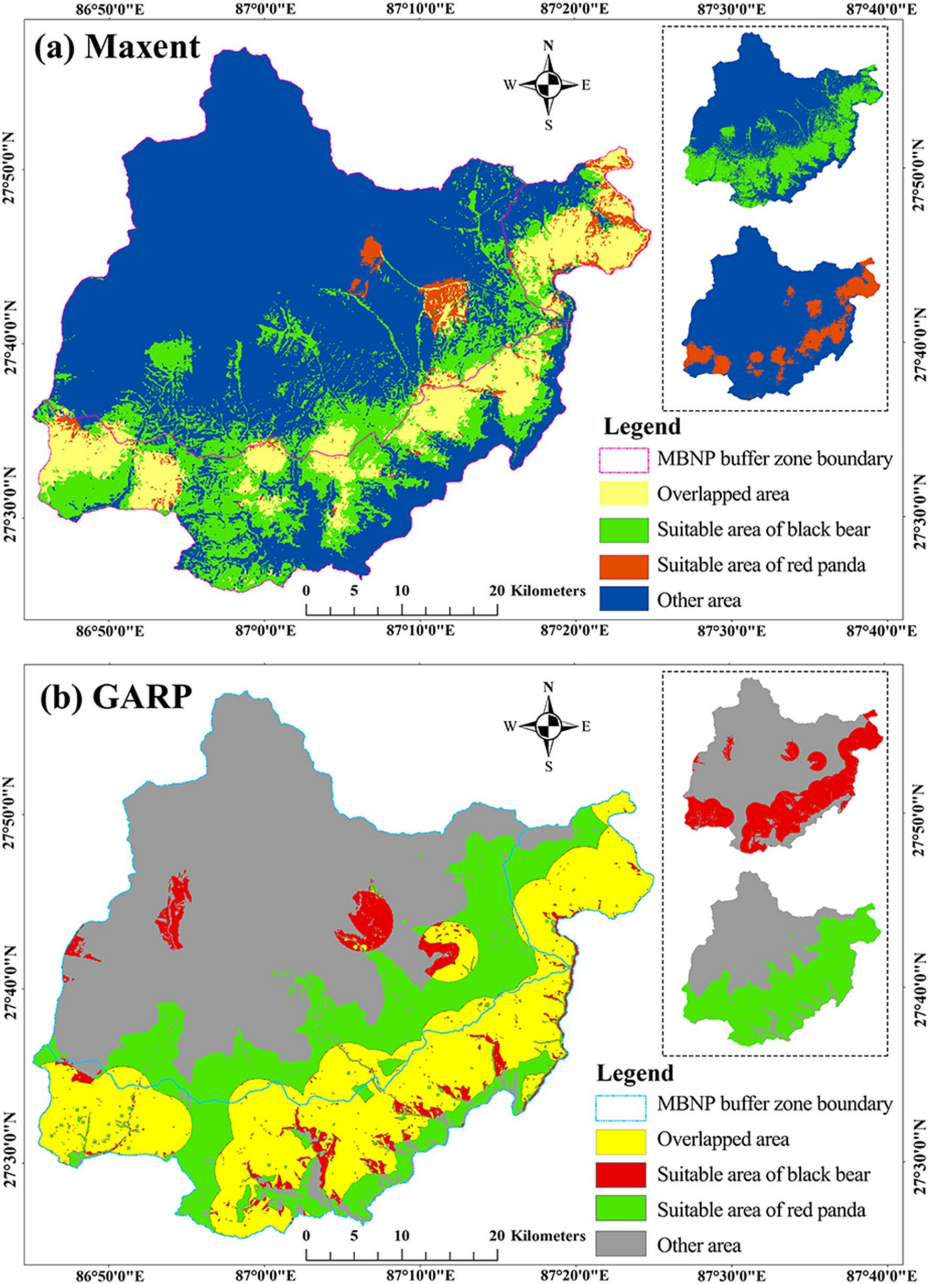
Predicted suitable habitat and overlapping habitat of the black bear and red panda from Maxent (**a**) and GARP (**b**). Map created in ArcMap 10 of the Environmental System Resource Institute, Inc. (https://desktop.arcgis.com/zh-cn/arcmap/).

Figure 6 depicts the habitat suitability maps generated from Maxent and GARP for black bear, red panda and the overlapping habitat for both of the species. (1) The Maxent model predicted that the suitable habitat for the black bear and red panda in the whole study area was 716 km^2^ and 343 km^2^, respectively. The overlapping habitat between the species was 283 km^2^, which was 83% of the total suitable habitat of the red panda but only 40% of the entire suitable habitat for the black bear. Out of the whole suitable habitat, the buffer zone of MBNP covered 70% of the black bear habitat, 82% of the red panda habitat, and 88% of the area suitable for both of the species. The performance of this model for both species was satisfactory. (2) The GARP model predicted that the suitable habitat for the black bear and red panda in the whole study area was 1074 km^2^ and 714 km^2^, respectively. The overlapping habitat between the species was 627 km^2^, which was 88% of the overall suitable habitat of the red panda but only 58% of the total suitable habitat for the black bear. Out of the complete suitable habitat, the buffer zone of MBNP covered 65% of the black bear habitat, 79% of the red panda habitat, and 84% of the appropriate area for both species.

### Evaluation of the models

**Figure 7:**
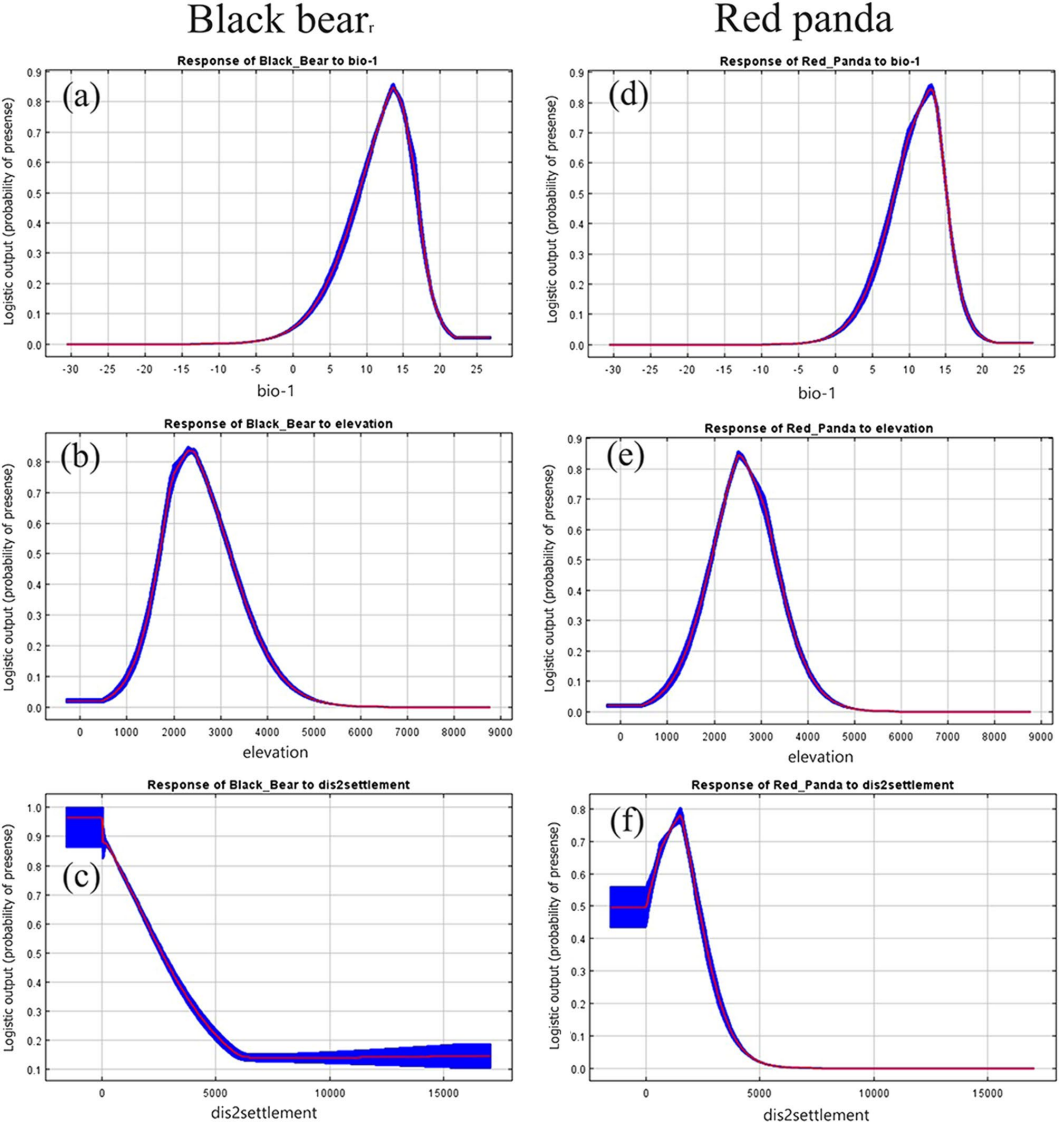
Response curves of selected variables for black bear (**a**, **b**, **c**) and red panda (**d**, **e**, **f**) habitat suitability in MBNP. The curves show the mean response of the 10 replicate Maxent runs (red) and the mean ± one standard deviation (blue, two shades for categorical variables).

Response curves display the relationship between habitat probability and bioclimatic variables. The response curves of mean annual temperature (Bio1) indicated that the optimal habitat for both the species was the area ranging between 10 and 15 ℃. The suitability of habitat dropped sharply as the temperature increased or decreased from this optimum. The tolerable range of heat in the suitable habitat for both species was from 0 to 20 ℃ (a & d in Fig. 7). Similarly, the relationship between the two species and elevation showed that the altitude between 2000 and 3000 m was the most suitable habitat range for these mammals. Both species can survive in a habitat that has an elevation that ranges from 1000 to 4500 m (b & e in Fig. 7). Another vital variable, distance to settlement, showed that these species were found within the range of 5 km from the nearest colony of people in the study area.

In the Maxent model for black bear, the average test AUC for the 10 replicate runs was 0.857 with a standard deviation of 0.036. For red panda, the average test AUC for the 10 replicate runs was 0.920, with a standard deviation of 0.022. In the GARP models, the AUC for the black bear with the best selection among the ten outputs of the subset was 0.791 and for the red panda, the average test AUC for the ten replicate runs was 0.786. The higher AUC of the Maxent model in comparison to GARP demonstrates the stronger prediction capability of Maxent. According to calculations of the pixel area of the predicted suitable habitat maps, the suitable regions predicted by GARP were much larger than the regions predicted by Maxent. The overlap of the suitable area predicted by the two models was calculated to investigate their agreement. It was found that the overlapping area between the models was slightly less than the total suitable area calculated by Maxent (Table 3).

**Table 3:** The difference in area calculated by Maxent and GARP for black bear and red panda in MBNP.

Species	Area (km^2^)			
	Maxent	GARP	Overlapped	Difference
Black bear	716	1074	658	358
Red Panda	343	714	329	371
Overlapped	283	627	273	344

## Discussion

### Suitable habitat coverage and overlapping

Analyzing the results from the two models, we find that the suitable area calculated by GARP is immensely greater than the area estimated by Maxent (Table 2 and Table 3). The suitable area projected by GARP for black bear is one and a half times greater than that by Maxent, and for the red panda, it is more than double (Table 3). Studies conducted on ecological niche modeling using these two models in India and Zimbabwe also resulted in larger suitable area prediction by GARP^67,68^ but not to the extent of this study. Unlike Maxent, which was explicitly developed for modeling presence-only species data and aimed at estimating the target distribution by finding the placement that approaches uniformity^13,69^, the GARP model uses both presence and pseudo-absence data of the species^20^. This might have led to an error in selecting the pseudo-absence points, which was thus reflected in false predicted areas for the species presence resulting in over prediction. Furthermore, the GARP models yielded lower AUC compared to the Maxent models for both black bear and red panda (< 0.8 for GARP and > 0.8 for Maxent), which shows that Maxent indicated better discrimination of suitable versus unsuitable areas for the species^9^.

The two different models showed that there is an intersection of a larger proportion of suitable habitat between these species. According to the calculations within Table 3, the distribution of red panda in Maxent and GARP respectively showed that 83% and 88% of the red panda distribution overlapped with the predicted black bear distribution. Black bear, which had a broader distribution, shared 40% from Maxent and 58% from GARP of the overlapping habitat with red panda. The pictorial distribution of these species in the map of Nepal by Jnawali et al.^70^ shows that the black bear has a much more extensive distribution than the red panda, which is highly in agreement with our mapped results. Panthi et al.^71^ estimated 18,193 km^2^ of suitable habitat for the red panda in Nepal while studies on suitable area calculation for black bear in Nepal are lacking. Sometimes, different models provide diverse predictions^72,73^, which was also observed in the current study. Another important facet to note is that the presence points of the black bear are evenly distributed across the study area when compared to that of the red panda (Fig. 2). The distribution of the presence points could have affected the overall prediction of the suitable habitat.

### Suitable habitat over land cover

In the suitable habitat maps obtained in the two models, the suitable habitat area in the national park buffer zone is more than that in the core area. The common habitat of both species in the buffer zone accounted for most of the area (over 80%) predicted by the two models (Fig. 6). It is evident from the land cover data (Table 2) that nearly two-thirds of the forest spreads into the buffer zone. Forest is the most preferred area for both of the species. More than 60% of the total forest of MBNP flourishes in the buffer zone of this national park. Himalayan black bear and red panda are sympatric mammal species that are found sharing the same habitat with similar features. They both live in temperate forests and also depend on similar food for survival^27,30,35,37^. Red pandas are habitat specialists, preferring to live under forests where there is a lot of bamboo. Among the vegetative related variables, this study was not able to distinguish the forest with bamboo and shrubs from the forest without bamboo, which is worthy of further improvement. Apart from the forest, bushy area and cultivation area are the other two major land covers that provide suitable habitat for these sympatric species. Both the models predict a range between 10 and 15% of suitable area for the two species lies in the cultivated area. This increases the probability for the animals to encounter anthropogenic activities and disturbances.

### Management and conservational implications

These species have been surviving under the threats induced by humans like poaching, retaliatory killing, proximity to herder’s sheds, tourist facilities, mismanagement of solid waste, consumption of human disposed of food, disturbance on habitat, etc^30,74–76^. Red panda prefers less disturbed areas to live in but will still occupy human troubled areas^77^. On the other hand, the black bear has encountered many conflicts with humans in different regions in Nepal and other territories across its range, including MBNP^43,78^. MBNP has been a promising tourist site with several trekking routes and tourist stations through the habitat of these animals^79^. At the same time, road extension activities are being carried out all over the buffer zone from Kimathanka in the East to Bung in the West. Though these kinds of development activities are symbols of improved infrastructure for local communities, they tend to cause destruction to the wildlife habitat. A population census of both of these species in MBNP has not been conducted so far. Though the actual population is unknown, development activities should be wildlife-friendly. There lies a risk for the endangered animal population, due to the habitat having a direct link with the animals’ wellbeing. Larger level studies at the national/regional scale are needed in order to generalize these results. Biodiversity is the basis for the survival and development of human society. For the survival and development of local environment, necessary measures should be taken to protect species and maintain biodiversity. Biotic interactions (e.g. predators or competitors) may have precluded a species from an otherwise suitable area. Historical geological factors may have hampered dispersal to certain areas and potentially represent a severe limiting factor in predictive models because they are not accounted for in the model^80^.This paper only predicted the habitat suitability of the two species and analyzed the overlapping areas, but did not consider the intra-specific competition and connectivity between habitats and population disequilibrium from the perspective of ecology^14^, which are also of important reference significance for the management and conservational implications of the two species in this region.

## Conclusion

This study presents the first application of two widely used SDMs for two mountainous area-dwelling mammal species of Nepal. We employed Maxent and GARP to map the suitable and overlapping habitat of Asiatic black bear and red panda in Makalu Barun National Park. The two models estimated the appropriate habitat area with somewhat different results. But the outcomes of these models on the overlapped area between the two species and preference of each animal on the different land cover were similar. Since different models have different results, hybrid or ensemble model frameworks could be used to make reliable and robust predictions of the potential distribution of species. The habitat for the study species stretches across the Southern part of the national park, covering most of the buffer zone area. We conclude that the suitable habitat of these sympatric species highly overlaps and forest is the land cover they prefer over other land use types. We suggest national park management, local governments, and other conservation partners preserve large areas of natural habitat for conservation of multiple species, especially potential overlapping habitats in the buffer zone to maintain biodiversity.
